# Relationship between empathic tendencies and trait anxiety among individuals interested in folk dances

**DOI:** 10.3389/fpsyg.2026.1901484

**Published:** 2026-08-05

**Authors:** Tuncay Öktem, Ramazan Akkuş, Ümit Öz, Umut Diyar Gök

**Affiliations:** 1 Faculty of Sports Science, Bayburt University, Bayburt, Türkiye; 2 Vocational School, Bayburt University, Bayburt, Türkiye; 3 Vocational School of Health Services, Bayburt University, Bayburt, Türkiye; 4 Bayburt University Faculty of Sports Science, Bayburt, Türkiye

**Keywords:** anxiety, dance, empathy, folk dances, physical education

## Abstract

**Introduction:**

Folk dances, which are among the most important cultural elements reflecting societies’ historical heritage, ways of life, and shared values, are regarded not only as an art form but also as a functional tool that contributes to an individual’s psychological well-being. Because they are based on physical activity and involve group participation, they can positively impact empathy and anxiety. The primary objective of this study is to examine the psychosocial characteristics of individuals who participate in folk dances, with a focus on the relationship between empathy and anxiety. In this context, folk dance will be discussed from various perspectives in relation to empathy and anxiety.

**Materials and methods:**

The study employed the correlational survey model, a quantitative research method. The study included 358 participants interested in folk dances. Data were collected using the Empathic Tendencies Scale and the trait anxiety section of the State-Trait Anxiety Inventory. Statistical analyses included descriptive statistics, a normality test, Pearson correlation, and regression analysis.

**Results:**

A negative, significant, and weak correlation was observed between participants’ trait anxiety and their empathic tendencies. Results from the regression analysis reveal that participants’ empathic tendencies significantly predict their trait anxiety, explaining 10.7% of the total variance.

**Conclusion:**

In conclusion, folk dance may be considered a factor associated with individuals’ anxiety and empathy levels.

## 1 Introduction

Culture is the sum of beliefs, acceptances, and ways of life that emerge from the depths of history, born from the needs of time and the conscious historical events, forming a meaningful and rich synthesis, systematically and unsystematically passed down from generation to generation, thereby leading to the acquisition of a sense of belonging and identity in each individual, giving them the power to change their environment and conditions, and developing a sense of reference to the past as generations look at their present and the future (Yaman, 2005). There are many elements connected to culture, among which folk dances stand out. It holds an important place among the countless forms of cultural heritage (Miao et al., 2025). It is also seen as a way of preserving cultural heritage (Javiña, 2020). As one of the most important cultural elements reflecting the historical accumulation, lifestyles, and shared values of societies, folk dances, as a symbolic existence representing cultural identity, are not only a concrete practice of the common experience of human groups, but also a concentrated embodiment of the historical memory and cultural heritage of a particular community (Chu, 2025). Reflecting a history that spans centuries and described as a manifestation of the emotions, events, pain, and joy—as well as happiness and suffering—those individuals experience in the world around them (Hua and Yang, 2025), folk dances incorporate a wide variety of local ethnic costumes and customs and are performed to the accompaniment of ethnic instruments that are rich in diversity and expression (Zhang, 2025). It refers to dance forms that preserve authentic folk taste and traditional characteristics (Miao et al., 2025). It can be considered a language that conveys information through body movements (Cai et al., 2023). It is also regarded as a form of cultural expression that combines physical activity, social interaction, and personal and emotional expression (Kapodistria et al., 2021). Folk dances, characterized by rhythmic movement patterns, are performed before audiences and demand high motivation, group cohesion, and advanced motor skills (Kaya, 2017). It is also known to significantly affect the acquisition of complex motor tasks, the reorganization of movement patterns, rhythmic coordination, precision, and balance (Marković et al., 2023). Based on this, it can be argued that folk dance encompasses a physical and athletic dimension rooted in movement and exercise, alongside its well-established cultural and artistic facets (Kaya, 2017).

Empathy is the ability to understand a person’s emotional states and intentions, to value their feelings, and to be sensitive to their needs, socially grounded in ability socialization (Srinivasan and González, 2022). Empathy, conceptualized as the capacity to understand and share another person’s emotional state while maintaining a clear distinction between the self and others (Eklund and Meranius, 2021), has long been recognized as a critical component of human social interaction (Crane, 2025). Empathy, described as a crucial skill vital for human interaction (Heyers et al., 2025), can also be characterized as a challenging and vulnerable trait (Polanowska and Krzeminska, 2026). Literature on empathy categorizes the construct into two primary dimensions: cognitive empathy, which entails the capacity to comprehend another individual’s emotional state, and emotional empathy, defined as the ability to provide an appropriate affective response to that state (Heyers et al., 2025). Empathic tendency constitutes the emotional dimension of empathy and refers to an individual’s potential to empathize (Karaca et al., 2013). Within the context of sports and physical education, empathy serves as a foundational cornerstone that fosters an inclusive environment, enabling all participants to develop and maximize their potential free from discrimination or inequitable treatment (Kalkan, 2022). Physical activity can be said to contribute to maintaining better cognitive empathy in healthy young adults (Shima et al., 2021b). Empathy, which plays a pivotal role in fostering an inclusive, supportive, and motivating environment (Lapanda et al., 2022), has also been demonstrated to enhance athletes’ mental resilience and athletic performance (Sepdanius et al., 2024b). Folk dances, characterized by structural demands for group performance, offer a distinctive environment that fosters interpersonal empathy. Being able to perform the same figure at the right time, with the right rhythm, and in harmony with others requires noticing, feeling, and positioning oneself accordingly. This process becomes a natural part of developing empathy skills. Therefore, it can be said that it can serve a function in increasing empathy levels.

Anxiety, defined as an individual’s state of emotional transition (Spielberger, 1972; cited in Martínez Valera et al., 2024), is an adaptive emotion critical for anticipating and preparing for future threats (Barlow, 2000; cited in Richardson, 2023). It is the most common mental health problem worldwide (Chellappa and Aeschbach, 2022). Because the social environment is constantly changing, and the pace of modern life is rapidly increasing (Xi, 2020). However, long-term anxiety can lead to psychological distress that affects an individual’s daily functioning (Cole, 2014). Participation in sports activities is known to facilitate positive mental health behaviors, including self-confidence, positive self-esteem, and social support (Armstrong and Oomen-Early, 2009). A review of the literature reveals that anxiety is one of the most frequently evaluated emotional variables in relation to an athlete’s skill in their sport (Ford et al., 2017). Several studies have found that physical activity is an effective way for individuals to reduce symptoms of depression and anxiety (Tyson et al., 2010; Wu et al., 2015; Mohammadi Orangi et al., 2021; LeBouthillier and Asmundson, 2017; Henriksson et al., 2022). It is also known that cultural and artistic activities based on physical activity are effective in reducing anxiety. Some studies on dance have shown it to be effective in reducing anxiety (Liu et al., 2023; Wang, 2022; Filippou et al., 2018, 2020; Zhang and Ma, 2025). In this context, the dual nature of folk dance, encompassing physical exercise and social togetherness, serves as an effective mechanism for alleviating anxiety.

Folk dance represents an activity grounded in harmonious interaction; it not only demands physical proficiency but also shapes intra-group socialization and facilitates the development of interpersonal communication skills. The group members’ capacity to move synchronously, sustain a shared rhythm, and exhibit mutual resilience in response to one another collectively contributes to enhancing this logical-empathic resilience. On the other hand, high levels of empathy optimize group communication and coordination by enabling individuals to remain highly perceptive of others’ emotional states and reactions. In addition, this situation can also make a person sensitive to social evaluation, performance expectations, and any negative feedback they may face. Theoretically, empathy is considered in two dimensions: cognitive and emotional. Cognitive empathy involves the mental recognition of others’ feelings and thoughts; conversely, emotional empathy shows the affective sharing of and susceptibility to those emotional experiences. Rather than segregating empathy into cognitive and emotional components, the Empathic Tendency Scale employed herein assesses an individual’s overall capacity to understand others’ emotional and cognitive states in daily life. In this context, it may be argued that empathy is associated with anxiety in settings characterized by intensive social interaction and synchronized group performance, such as folk dance. Empathic tendencies may enable individuals to better understand their peers’ emotional states and manage the social pressures associated with group performance, thereby contributing to the relationship between empathy and anxiety.

## 2 Materials and method

### 2.1 Research design

The study employed the correlational survey model, a quantitative research method. The correlational survey design is an approach that aims to identify the relationship or effect between two distinct quantitative variables (Fraenkle et al., 2012) and determine the degree of this association (Karasar, 2015).

### 2.2 Research aim

The primary aim of this study is to examine the psychosocial characteristics of individuals participating in folk dance by focusing on the relationship between empathy and anxiety. A secondary aim is to demonstrate that, beyond its value as a form of traditional cultural heritage, folk dance may also serve as a mechanism associated with individual psychological well-being. In line with this aim, the following research questions are addressed:

1. Does a significant difference exist in participants’ empathic tendency levels based on gender?
2. Do participants’ levels of trait anxiety differ according to gender?
3. Do participants’ levels of empathic tendencies differ depending on their participation in the competition?
4. Do participants’ trait anxiety levels differ depending on their participation in the competition?
5. Do participants’ empathic tendencies differ according to the length of time they have been involved in folk dance?
6. Do participants’ trait anxiety levels differ depending on the length of time they have been involved with folk dances?
7. Is there a relationship between participants’ empathic tendencies and their trait anxiety levels?
8. Do participants’ empathic tendencies influence their trait anxiety levels?

### 2.3 Sample size

In this study, the sample size was determined by applying power analysis. The minimum required sample size was determined using the effect size guidelines proposed by Cohen (1992). An *a priori* power analysis revealed that a total of 314 individuals was required to ensure sufficient statistical power. A sample size of 300 or more is categorized as “good” for ensuring factor stability (Comrey and Lee, 1992). After accounting for potential participant attrition, the final target sample size was set at 400 participants. Following the exclusion of incomplete or incorrectly completed questionnaires, the final sample consisted of 358 participants.

### 2.4 Participants

The participants in this study are individuals from the province of Bayburt in Türkiye who regularly practice folk dances, attend rehearsals, take part in performances and competitions, and are actively involved in this field. Folk dances, a vibrant tradition that reflects the emotional world, values, and cultural heritage of Bayburt society from the past to the present, are one of the most prominent elements of the cultural identity of the people of Bayburt. They serve as a powerful means of expression reflecting the historical accumulation, social structure, and shared memory of this ancient city. The rigidity, discipline, and harmony seen in the dance figures offer important clues about the geographical conditions, historical struggles, and lifestyle of the region. The study sample consisted of 358 participants residing in the province of Bayburt, Türkiye, who were involved in folk dance. Participants were contacted between October 6, 2025, and December 26, 2025. Participants were recruited through convenience sampling from volunteers who were available during the data collection period. Before data collection, participants were informed about the study, provided electronic informed consent, and were assured that their participation was voluntary. Data were collected using an online questionnaire.

#### 2.4.1 Inclusion criteria

The inclusion criteria were being involved in folk dance, voluntary participation, and completion of the survey in its entirety.

#### 2.4.2 Exclusion criteria

Not being interested in folk dances and giving incomplete or incorrect answers were determined as exclusion criteria from the study.

### 2.5 Data collection tools

#### 2.5.1 Personal information form

The form was developed to collect participants’ demographic and contextual characteristics, including gender, age, marital status, education level, duration of involvement in folk dance, and competition participation.

#### 2.5.2 Empathic tendency scale (ETS)

Developed by Dökmen (1988), this scale was designed to measure individuals’ empathic tendencies. In the validity and reliability study of the scale, the Cronbach’s alpha internal consistency coefficient was reported as 0.72. The scale is a 5-point Likert-type instrument consisting of 20 items. Responses are scored as follows: 1 = completely inappropriate, 2 = mostly inappropriate, 3 = undecided, 4 = mostly appropriate, and 5 = completely appropriate. Items 3, 6, 7, 8, 11, 12, 13, and 15 are reverse-scored. The total score ranges from 20 to 100, with higher scores indicating higher levels of empathic tendency. In the present study, the Cronbach’s alpha internal consistency coefficient was calculated as 0.747.

#### 2.5.3 State-trait anxiety inventory (STAI)

This is a scale developed by Spielberger et al. (1970) to determine a person’s situational and trait anxiety levels. The Turkish translation, reliability, and validity study were also conducted by Öner and Le Compte (1985). It consists of 40 items made up of short statements. The scale consists of two parts: a 20-item “situation anxiety form” designed to determine feelings experienced at the moment, and a 20-item “trait anxiety form” designed to determine feelings experienced over the past 7 days. The scale, which is a 4-point Likert type, is reported to have an Alpha reliability ranging from 0.83 to 0.87, a test-retest reliability ranging from 0.71 to 0.86, and an item reliability ranging from 0.34 to 0.72 (Öner and Le Compte, 1985). In this study, the trait anxiety dimension of the scale was used. In this study, the Cronbach’s Alpha internal consistency coefficient, obtained as a result of the validity and reliability study of the scale, was determined to be 0.675. The internal consistency reliability coefficient of the Trait Anxiety Scale in the current sample was α = 0.675, indicating a moderate level of reliability. Although this value provides an acceptable basis for interpreting the findings, it should be acknowledged as a limitation of the present study. The relatively modest reliability coefficient suggests that measurement-related factors may have influenced the precision of the trait anxiety scores.

### 2.6 Data analysis

The IBM SPSS Statistics 25 software package was used for data analysis. The quantitative data obtained were first tested for normality (Table 1). The data were found to have skewness and kurtosis values ranging from −1.5 to +1.5. A review of the literature shows that this range represents a normal distribution (Tabachnick and Fidell, 2013). After confirming that the normality assumption was met, parametric statistical tests were applied. The findings were evaluated at a significance level of 0.05 within a 0.95 confidence interval. The assumptions of linear regression analysis were tested prior to the analysis. The examinations showed that the assumptions of linearity, homoskedasticity, independence of errors, and normality of residuals were met. Additionally, Cook’s distance, leverage values, and standardized residuals were examined; no significant outliers were identified that would affect the analysis.

**TABLE 1:** Skewness and kurtosis values for scales.

Variable	*N*	$\bar{\text{x}}$	Sd	Skewness	Kurtosis
Scales					
Empathic tendency scale	358	3.1418	0.33698	0.633	0.752
Trait anxiety inventory	358	2.3390	0.32649	−0.709	0.246

As shown in the table, the skewness and kurtosis values of both scales were found to range between −1.5 and +1.5. Tabachnick and Fidell (2013) state that skewness and kurtosis values in the range of −1.5 to +1.5 indicate a normal distribution. Based on these findings, it can be concluded that the data met the assumption of normality.

### 2.7 Ethical statement

This study adheres to research ethics principles and has obtained the necessary ethics committee approvals. As part of the ethics committee approval, a document was obtained from the Bayburt University Ethics Committee dated 07.05.2025, with decision number 189 and document number E-51694156-050.04-277753. All participants were provided with detailed information about the purpose, procedures, and scope of the research, and electronic informed consent was obtained from each participant before participation. Participation in the study was entirely voluntary, and participants were informed that they had the right to withdraw from the study at any stage without any penalty or disadvantage. The confidentiality and anonymity of participants’ personal information and responses were ensured throughout the research process, and all data were handled and reported in accordance with ethical research standards.

## 3 Results

Table 2 illustrates the detailed findings. The average age of the participants was 23.82 ± 4.017; according to the gender variable, 126 (35.2%) were female and 232 (64.8%) were male; according to the marital status variable, 67 (18.7%) were married and 291 (81.3%) were single; according to the education level variable, 15 (4.19%) had middle school education, 104 (29.05%) had high school education, 60 (16.76%) had associate’s degrees, 178 (49.72%) had bachelor’s degrees, and 1 (0.28%) had a master’s degree; according to the length of experience in folk dance, 170 (47.49%) had been involved for up to 5 years, 129 (36.03%) for 5–9 years, and 59 (16.48%) for 10 years or more; and according to their participation in competitions; It was determined that 211 of them (58.9%) had previously participated in a competition, while 147 of them (41.1%) had not participated in any competition.

**TABLE 2:** Information regarding the demographic characteristics of the participants.

Variable		*N*	%
Gender			
	Female	126	35.2
	Male	232	64.8
Marital status			
	Married	67	18.7
	Single	291	81.3
Education level			
	Middle School	15	4.19
	High school	104	29.05
	Associate degree	60	16.76
	Bachelor’s degree	178	49.72
	Master’s degree	1	0.28
Duration of folk dance experience			
	Up to 5 years	170	47.49
	5–9 years	129	36.03
	10 years and over	59	16.48
Competition participation status			
	Yes	211	58.9
	No	147	41.1

The analysis of the findings in Table 3 reveals a statistically significant difference (*p* < 0.05) in the trait anxiety scale in favor of males, and in the empathic tendency scale in favor of females.

**TABLE 3:** Independent samples *T*-test analysis of the empathic tendency scale and trait anxiety inventory based on gender.

Scales	Gender	*n*	$\bar{\text{x}}$	Sd	t	*P*
Trait anxiety	Female	126	2.2393	0.34326	−4.211	**<0.001**
	Male	232	2.3931	0.30433		
Empathic tendency	Female	126	3.2821	0.36892	5.694	**<0.001**
	Male	232	3.0655	0.29199		

The bold values indicate the *p* < 0.001.

An analysis of the findings in Table 4 reveals a statistically significant difference on the trait anxiety scale in favor of the participants who took part in the competition (*p* < 0.05). However, no statistically significant difference was observed on the empathic tendency scale (*p* > 0.05).

**TABLE 4:** Independent samples *T*-test analysis of the empathic tendency scale and trait anxiety inventory based on participants’ competition participation status.

Scales	Eligibility to participate in the competition	*n*	$\bar{\text{x}}$	Sd	t	*P*
Trait anxiety	Yes	211	2.3055	0.32976	−2.342	**0.019**
	No	147	2.3871	0.31667		
Empathic tendency	Yes	211	3.1540	0.34382	0.832	0.406
	No	147	3.1241	0.32726		

The bold value indicates the *p* < 0.050.

As shown in Table 5, a one-way analysis of variance (ANOVA) was conducted to examine whether participants’ trait anxiety levels differed significantly based on their duration of involvement in folk dance. The results showed no statistically significant difference (*p* > 0.05). On the Empathic Tendency Scale, a statistically significant difference was found between the “10 Years and Over” and “Up to 5 Years” groups, in favor of the “10 Years and Over” group (*p* < 0.05).

**TABLE 5:** ANOVA test on scores on the empathic tendency scale and the trait anxiety inventory according to the variable of participants’ duration of folk dance experience.

Scales	Duration of folk dance experience	*N*	$\bar{\text{x}}$	Sd	F	*p*	*Post-hoc*
Trait anxiety	Up to 5 years (a)	170	2.3150	0.32987	0.875	0.418	–
	5–9 years (b)	129	2.3620	0.32445			
	10 years and over (c)	59	2.3576	0.32174			
	Total	358	2.3390	0.32649			
Empathic tendency	Up to 5 years (a)	170	3.1832	0.34397	3.228	**0.041**	c > a
	5–9 years (b)	129	3.1244	0.33061			
	10 years and over (c)	59	3.0602	0.31701			
	Total	358	3.1418	0.33698			

The bold value indicates the *p* < 0.050.

Table 6 shows a negative, significant, and weak correlation between participants’ trait anxiety and their empathic tendencies (r: −0.328, *p* < 0.005). The correlation coefficient between the two variables is as follows: R(356) = −0.328 *p* < 0.001 95% CI [−0.417, −0.232].

**TABLE 6:** Correlation analysis of the empathic tendency scale and the trait anxiety inventory.

Variable		Trait anxiety
**Empathic** t**endency**	r	−0.328**
	*p*	**<0.001**

**p < 0.01. The bold value indicates the *p* < 0.001.

When the regression analysis results in Table 7 are examined, it is seen that empathic tendency is a significant predictor of trait anxiety, *R* = 0.328, R^2^ = 0.107, F(1, 356) = 42.860, *p* < 0.01. It can be stated that 10.7% of the trait anxiety of the participants interested in folk dances is explained by their empathic tendency. The unstandardized regression coefficient is as follows: *B* = −0.318, SE = 0.049, *t* = −6.547, *p* < 0.001. The standardized beta is as follows: (β = −0.328, *p* < 0.001). The confidence intervals for R^2^ are as follows: F(1, 356) = 42.86, *p* < 0.001, R^2^ = 0.107, 95% CI [0.057, 0.165].

**TABLE 7:** Regression analysis of the prediction of participants’ trait anxiety levels based on their empathic tendencies.

Variable	B	Standard error	β	t	*p*
Constant	3.337	0.153	–	21.768	**<0.001**
Empathic tendency	−0.318	0.049	−0.328	−6.547	**<0.001**

The bold values indicate the *p* < 0.001.

## 4 Discussion and conclusion

When the results of the independent samples *t*-test according to the gender variable of the participants (Table 3) were examined, it was determined that the anxiety levels of male participants were higher than those of female participants. While no studies on trait anxiety levels among individuals involved in folk dances were found in the literature, numerous studies based on physical activity and sports have reached different conclusions. In the study conducted by Özgül (2003) examining the continuous anxiety levels of physical education and sports college students, a difference was found in favor of female students. In a study conducted by Yüksel and Kaya (2023) on security personnel who exercise, a difference was found in favor of female participants. In a study conducted by Yılmaz et al. (2024) on the anxiety levels of adolescent individuals who participate in sports, a difference was found in favor of female participants. In the study conducted by Yücel (2003) on taekwondo athletes, no difference was found in terms of the gender variable. In the study conducted by Acharjee et al. (2025) to determine the anxiety levels experienced by athletes, no difference was found in terms of the gender variable. In the study conducted by Öktem et al. (2020) on the anxiety levels of boxers who are candidates to participate in the Olympic Games, no difference was found in terms of the gender variable. General gender differences observed at the overall level mask significant differences depending on the type of activity: some sports are practiced predominantly by men, while others are almost entirely practiced by women (Chalabaev et al., 2013). Gender differences exist in participation in physical activity, and these differences are most pronounced in gender-stereotyped activities such as soccer and dance (Anderson et al., 2017). Studies in the literature show that dance is effective in improving the quality of life and reducing clinical symptoms such as depression and anxiety (Mishra and Shukla, 2022). Dance is a popular form of physical exercise, and studies clearly show that it can reduce anxiety, increase self-esteem, and improve psychological well-being (Maraz et al., 2015). According to the current literature, women have participated more in dance activities than men (Barna et al., 2023). In the study by Zaletel and Kajtna (2020), it was found that female dancers placed more importance on socially oriented reasons, as well as reasons such as self-control, emotional relief, and self-awareness, compared to male dancers. When the results obtained are evaluated in light of the information provided in the literature, it can be stated that the cultural role expectations of male participants and the emotional flexibility of female participants in folk dances from the Bayburt region are the cause of the sample-related result.

When the results of the independent samples *t*-test according to the gender variable of the participants (Table 3) were examined, it was determined that the empathic tendencies of female participants were higher than those of male participants. While no studies specifically addressing the empathic tendencies of individuals involved in folk dances were found in the literature, there are studies that have reached similar but different conclusions based on physical activity and sports. Koçak and Balçıkanlı (2021) determined that there is a difference in empathic thinking skills in favor of women among elite athletes who practice martial arts. In a study by Lorimer and Jowett (2010) titled “The Effect of Role and Gender on Empathic Accuracy of Coaches and Athletes,” it was determined that female athletes exhibited higher empathic accuracy than male athletes. Löffler and Greitemeyer (2023) state that females tend to give stronger empathetic responses than males. In the study conducted by Paro et al. (2014), it was found that female medical students tended to empathize more than male students. Research on sports shows that male and female participants differ in their empathy (Sevdalis and Raab, 2014). Generally, females are portrayed as more compassionate and empathetic, while males are depicted as less emotional and more cognitive (Christov-Moore et al., 2014). In this context, it is believed that the fact that female participants are compassionate and easily form bonds, combined with the wordless and collective movement aspect of folk dances, increases their sensitivity to the emotional world of others. This situation can be attributed to the fact that the female participants acted in a way that supported or maintained team spirit within the folk dance team.

When the results of the independent samples *t*-test (Table 4) were examined according to the participants’ participation status in the competition, it was determined that individuals who did not participate in the competition had higher anxiety levels than those who did participate. No differences were found in the participants’ empathic tendencies. Self-confidence and anxiety are important variables that form the basis of the flow experience in sports (Koehn, 2013). Specifically, previous research has demonstrated that self-confidence is positively correlated with flow, whereas anxiety is negatively correlated with flow (Koehn, 2013). It can be stated that athletes experience different levels of anxiety during the competition process and that athletes with different competition results give different importance to anxiety (Zhang, 2023). Studies show that athletes with stronger psychological resilience naturally tend to pursue challenging goals rather than goals stemming from a fear of failure. Clarifying these goal-setting patterns can help athletes better understand and regulate their cognitive and physical anxieties in competitions (Aydın, 2025). Based on an examination of the findings in light of the existing literature, differences in anxiety levels were observed between individuals who participate in competitions and those who do not, with competition participation being associated with anxiety levels.

When the results of the independent samples *t*-test (Table 5) were examined according to the experience levels of the participants, it was determined that individuals with 10 years or more of experience had higher levels of empathy than those with up to 5 years of experience. No differences were found in the anxiety levels of the participants. Empathy plays a critical interpersonal and social role, acting as an emotional bridge that enables the sharing of experiences, needs, and desires between individuals and supports social behaviors (Riess, 2017). Shima et al. (2021a) state that sports experience can contribute to the development of empathy in people. Based on these findings, individuals with longer experience in folk dance tend to report higher levels of empathy.

When the relationship between participants’ levels of trait anxiety and their empathic tendencies was examined (Table 6), a negative, significant, and weak correlation was observed between the variables (r: −0.328, *p* < 0.005). Anxiety, a negative emotional state that arises in real or perceived threat situations (Eysenck et al., 2007), alters an individual’s tendency to cope with threats, causing the activity of the autonomic nervous system to appear in an excited state (Battalio et al., 2020). The way an individual uses their mind to interpret a physically stimulating situation can have a strong influence on how they will perform in that situation (Maloney et al., 2014). Empathy can be defined as a person’s ability to put themselves in another’s place and thus understand their feelings, thoughts, and attitudes (Fernandez and Zahavi, 2020). This socio-emotional reflex, triggered by perceiving another person’s emotional state, is a fundamental component of social interactions (Decety et al., 2015). It can be said that there is a close relationship between emotion regulation (McRae and Gross, 2020), which involves attempts to influence one’s own or others’ emotions, and empathy, and that emotion regulation increases empathy. In the fields of sports, exercise, and performing arts, the aim of understanding empathy is to improve performance at the perceptual-motor level on the one hand, and to encourage performers to enrich their potential at the social-emotional level on the other (Sevdalis and Raab, 2014). In the study conducted by Tang et al. (2024), a negative relationship was found between the anxiety levels and empathy levels of the participants. Gambin and Sharp (2018) suggest that empathy-related processes may play a role in the development or maintenance of anxiety symptoms. In contrast, Jütten et al. (2019) suggest that less emotional empathy is associated with fewer anxiety symptoms. In the study by Rodrigues Sampaio et al. (2020), it is suggested that empathy may be related to mental health. Based on these findings, it may be suggested that the emotional regulation capacity of individuals with higher levels of empathy who participate in folk dance represents one of several factors that may be associated with the relationship between empathy and anxiety.

When the findings regarding the effect of participants’ empathic tendencies on their levels of trait anxiety are examined (Table 7); according to the regression analysis results, empathic tendency is seen to be a significant predictor of trait anxiety (*R* = 0.328, R^2^ = 0.107, F(1, 356) = 42.860, *p* < 0.01). It can be stated that 10.7% of the trait anxiety of participants who are interested in folk dances is explained by their empathic tendencies. This finding shows that empathic tendency has a statistically significant but relatively limited effect on trait anxiety. It is known that folk dance practices have an exercise quality (Gerek, 2007). Physical activity can also be associated with physical health, psychological well-being, and social interaction skills (Çakır et al., 2025). Participating in sports can contribute to individuals not only physiologically but also psychologically, and in this respect, it can have positive effects on people’s psychology, such as happiness (Öktem, 2022). When the literature is examined; Numerous studies have shown that physical activity contributes to the development of empathy (Shima et al., 2021b), that empathy in sports activities increases the mental resilience and performance of athletes (Sepdanius et al., 2024a), that physical activity has a positive effect on alleviating anxiety in individuals (Lin and Gao, 2023), that participation in physical activity is protective against anxiety symptoms and disorders (McDowell et al., 2019), that individuals who regularly engage in physical activity have lower anxiety levels (Alves et al., 2019), and that it is associated with a reduction in anxiety symptoms in the general population (Kandola et al., 2018). A systematic review by Derksen et al. (2012) states that empathy is effective in reducing anxiety. A study by Chen et al. (2024) found that high levels of empathy positively affect mental health. Based on the results obtained and information from the literature, the study shows that the empathy level of individuals involved in folk dances plays a statistically significant and somewhat predictive role in the tendency toward continuous anxiety. Therefore, it can be argued that, particularly in the context of folk dances, empathetic tendencies can be considered a psychosocial variable that may influence individuals’ emotional processes and anxiety levels.

According to the results of the study, it was determined that females involved in folk dances have lower trait anxiety levels and higher empathic tendencies compared to males. Individuals participating in competitions were found to have lower trait anxiety levels than those who did not participate. It was also determined that the empathic tendencies of individuals involved in folk dances increased as their experience length increased. According to the correlation analysis results obtained from the study, a negative association was observed between empathy levels and anxiety levels among individuals involved in folk dance; however, the low correlation coefficient indicates that the relationship between these variables is limited. According to the regression analysis results obtained from the study, it can be said that as the level of empathy increases, a change occurs in the level of anxiety, and empathy has a significant effect on anxiety. In conclusion, the findings suggest that engagement in folk dance may be associated with trait anxiety levels and empathic tendencies among individuals. Within the scope of this study, it can also be suggested that trait anxiety and empathic tendencies are related constructs. The recommendations presented to institutions and researchers are as follows:

- It is recommended that training practices on empathy and anxiety be added to the curricula of institutions affiliated with the Ministry of National Education and the Higher Education Council.
- Conferences, talks, etc., on empathy and anxiety can be organized to convey this information to individuals.
- It is recommended that research be conducted that includes other psychosocial variables that may have an effect on empathy and anxiety.
- The study was limited to individuals residing in Bayburt province and involved in folk dances. It is recommended that future studies be conducted on larger sample sizes.
- The scarcity of studies in the field of sports science regarding the effect of empathy levels on anxiety levels is noteworthy. Increased research in this area is recommended.

## Data Availability

The raw data supporting the conclusions of this article will be made available by the authors, without undue reservation.
